# The Roles of miR-26, miR-29, and miR-203 in the Silencing of the Epigenetic Machinery during Melanocyte Transformation

**DOI:** 10.1155/2015/634749

**Published:** 2015-11-04

**Authors:** Cláudia Regina Gasque Schoof, Alberto Izzotti, Miriam Galvonas Jasiulionis, Luciana dos Reis Vasques

**Affiliations:** ^1^Department of Biochemistry, Federal University of Sao Paulo, 04044-020 Sao Paulo, SP, Brazil; ^2^Department of Genetics and Evolutionary Biology, University of Sao Paulo, 05508-090 Sao Paulo, SP, Brazil; ^3^Department of Health Sciences, University of Genoa, 16132 Genoa, Italy; ^4^IRCCS AOU San Martino IST, 16132 Genoa, Italy; ^5^Department of Pharmacology, Federal University of Sao Paulo, 04039-032 Sao Paulo, SP, Brazil

## Abstract

The epigenetic marks located throughout the genome exhibit great variation between normal and transformed cancer cells. While normal cells contain hypomethylated CpG islands near gene promoters and hypermethylated repetitive DNA, the opposite pattern is observed in cancer cells. Recently, it has been reported that alteration in the microenvironment of melanocyte cells, such as substrate adhesion blockade, results in the selection of anoikis-resistant cells, which have tumorigenic characteristics. Melanoma cells obtained through this model show an altered epigenetic pattern, which represents one of the first events during the melanocytes malignant transformation. Because microRNAs are involved in controlling components of the epigenetic machinery, the aim of this work was to evaluate the potential association between the expression of *miR-203, miR-26*, and *miR-29* family members and the genes *Dnmt3a, Dnmt3b, Mecp2*, and *Ezh2* during cells transformation. Our results show that microRNAs and their validated or predicted targets are inversely expressed, indicating that these molecules are involved in epigenetic reprogramming. We also show that miR-203 downregulates Dnmt3b in mouse melanocyte cells. In addition, treatment with 5-aza-CdR promotes the expression of *miR-26* and *miR-29* in a nonmetastatic melanoma cell line. Considering the occurrence of CpG islands near the *miR-26* and *miR-29* promoters, these data suggest that they might be epigenetically regulated in cancer.

## 1. Introduction 

Epigenetic modifications play a pivotal role in modulating gene expression during development and tissue differentiation, in the establishment and maintenance of genomic imprinting, and in X chromosome inactivation in female mammals. The most commonly studied type of epigenetic modification is the DNA methylation of CpG dinucleotides. In normal human cells DNA methylation typically occurs throughout the genome and at repetitive DNA, preserving genomic stability and inhibiting unwanted transposon reactivation [1–3]. CpG-rich regions, known as CpG islands [4], which are localized near 60% of coding gene promoters, are hypomethylated in normal cells [5]. Only one-tenth of these CpG islands are methylated in a tissue-specific manner [6].

DNA methylation is regulated through a family of DNA methyltransferases (Dnmt) comprising 5 members. Three of these proteins mediate the addition of a methyl group predominantly to DNA cytosines of CpG dinucleotide sequences. Dnmt1 is ubiquitously expressed at high levels in proliferating cells and is involved in the maintenance of DNA methylation. Dnmt3a and Dnmt3b promote* de novo* DNA methylation and are highly expressed at the early stages of development and also in embryonic stem cells (ESC). Immediately after differentiation, both proteins are downregulated and then remain ubiquitously expressed at low levels in somatic cells [7].

Posttranslational histone modifications are also involved in gene regulation, as the acetylation, methylation, or phosphorylation of histones can influence their affinity for DNA. Depending on the specific combination of these marks, they can either facilitate (e.g., histone 3 lysine 4 trimethylation, H3K4me3; histone 3 lysine 9 acetylation, H3K9ac) or impede (e.g., H3K9me3; H3K27me3) the binding of transcriptional factors to promoter regions [8–10]. DNA methylation and histone modifications are associated through methyl-binding proteins (MBP). The best-known MBP, Mecp2, contains two domains: one that specifically recognizes methylated DNA, a methyl-binding domain (MBD) [11]; and another that binds to Sin3 recruiting histone deacetylases, a transcriptional repression domain (TRD) [12–14] promoting gene silencing. Despite being ubiquitously expressed throughout all human tissues,* MECP2* is highly expressed in the brain [15].

Contrasting observations have been made in tumor cells, where repetitive DNA is globally demethylated, promoting genomic instability [16–18], and oncogene promoters are less frequently methylated, leading to aberrant expression [19]. However, a myriad of methylated CpG islands near promoters, many of which found in tumor suppressor genes [20], have been observed in cancer cells [21], and the hypermethylation profile depends on the tumor type [22–24]. The pattern of posttranslational histone modifications is also disrupted in tumor cells and might play an essential role in tumor initiation [25]. As an evidence of how important epigenetic alterations are during the early steps of malignant transformation, a recent study showed that murine melanocyte cells that are resistant to apoptosis induction by adhesion blockade (anoikis-resistant cells) could be selected when cultured in agarose-coated plates. The clonal colonies obtained after sequential cycles through the same process were reported as tumorigenic when injected in syngeneic mice [26]. However, when cells were treated with 5-aza-2′-deoxycytidine, a demethylating agent, before each cycle of anchorage blockade, the derived cells were not malignantly transformed. Therefore, this study shows that most epigenetic modifications occur as primordial and essential events during the establishment of malignant transformation in this murine melanoma model, rather than as a consequence of cell transformation [27]. Since microRNAs (miRNAs) are predicted to regulate the expression of 60% of all coding genes [28] including the epigenetic machinery (reviewed in [29]), they are good candidates for promoting aberrant expression of epigenetic machinery, contributing to the deregulation of epigenetic marks during cell transformation.

Here, using this murine melanoma model [26, 27], we demonstrate that Dnmt3a and Dnmt3b are downregulated in nonmetastatic (4C3−) and metastatic (4C3+) melanoma cell lines, concomitant with the overexpression of* miR-29b* and* miR-29c*, which are known* Dnmt3a* and* Dnmt3b* regulators [30].* miR-26a*, which targets Ezh2 [31], a histone methyltransferase belonging to the Polycomb family, was found to be downregulated in a nonmetastatic melanoma cell line (4C3−) but upregulated in 4C3+. These data are consistent with the fluctuation of* Ezh2* expression during the transition from a nonmetastatic to a metastatic melanoma phenotype. Moreover, the members of the* miR-26* family potentially target Mecp2 and Dnmt3b mRNAs, as predicted by several* in silico* miRNA target prediction programs, are upregulated in 4C3+ cells, whereas* Mecp2* and* Dnmt3b* were downregulated. Indeed, recent studies have shown that transfection of miR-26b in breast cancer cells showed diminished expression of DNMT3B. On the other hand, transfection of miR-26b antagomiR showed an overexpression of DNMT3B [32], suggesting that this miRNA is involved in DNMT3b regulation.

We have also seen an inverse correlation between this miR-203 and DNMT3b in nonmetastatic melanoma cells compared to normal melanocytes. This result is in agreement with the predicted target of miR-203 and with the findings of Coleman group that have reported that breast cancer cells with hypermethylated DNA phenotype have DNMT3b overexpression and a downregulation of miR-203 [32, 33]. In order to validate Dnmt3b as miR-203 target, we have transfected cell with miR-203 expressing vector, which promotes Dnmt3b downregulation. Additionally, miR-203 is also predicted to target Mecp2, showing an inverse correlation of expression between normal melanocytes and nonmetastatic melanoma.

Furthermore, to determine whether* miR-26*,* miR-29* family members and miR-203 could be epigenetically regulated, cells were treated with the hypomethylating agent 5-aza-deoxycytidine (5-aza-CdR). The results showed increased expression of all of the studied miRNAs in 4C3− cells, which might suggest that these miRNAs are epigenetically regulated and are more susceptible to destabilization during cell transformation. Taken together, these data suggest that epigenetic alterations that occur early during malignant transformation might be a result from the modulation of* miR-26*,* miR-29, miR-203,* and the consequent effects on key genes involved in the epigenetic machinery.

## 2. Materials and Methods

### 2.1. Cell Culture, Treatment with 5-aza-CdR, and Transfection with miR-203

Nonmetastatic (4C3−) and metastatic (4C3+) murine melanoma cells were obtained from melan-a nontumorigenic melanocytes [34] through repetitive anchorage blockade cycles, as described by Oba-Shinjo et al. [26]. Melan-a, 4C3−, and 4C3+ cells were grown in RPMI medium, pH 6.9 (Gibco, Carlsbad, CA), supplemented with 5% fetal bovine serum (Vitrocell, Campinas, SP, Brazil) and 1% penicillin-streptomycin solution (Life Technologies, Carlsbad, CA) at 37°C under 5% CO_2_. In addition, phorbol myristate acetate (PMA, Sigma, St. Louis, MO) was added at a final concentration of 200 nM to the complete medium for melan-a cells. The cells were treated with 10 *μ*M 5-aza-deoxycytidine (5-aza-CdR, Sigma, St. Louis, MO) for 48 hours, followed by RNA or total protein extraction.

An aliquot of 3,5 *μ*g of PSilencer 4.1-CMV-Puro plasmid containing the sequence of mmu-miR-203 or scrambled was kindly provided by Dr. Candi, Faculty of Medicine, Department of Experimental Medicine and Surgery, Rome [35]. They were transfected into cells with Fugene 6 (Promega), and culture was maintained in medium with 4 ug/mL puromycin through 14 days in order to select transfected resistant cells, followed by RNA and total protein extraction.

### 2.2. Methylation-Sensitive Single-Nucleotide Primer Extension Assay (Ms-SNuPE)

Global methylation status was inferred by methylation-sensitive single nucleotide primer extension (Ms-SNuPE) method. As previously described [27], the average of methylated CpG sites located at two different repetitive elements (retrovirus-C long terminal repeats, RC-LTR, and intracisternal A-particle elements, IAPy) was performed.

### 2.3. Retrotranscription and Quantitative Real-Time PCR

Total RNA extracted using TRIzol (Life Technologies, Carlsbad, CA) was treated with DNase I (Promega, Fitchburg, Wisconsin) and retrotranscribed using the High Capacity cDNA Reverse Transcription Kit (Life Technologies, Carlsbad, CA), and the corresponding loop-primer of the miRNA Assay Kit (Life Technologies, Carlsbad, CA), according to the manufacturer's instructions. The reverse transcription reaction was followed by quantitative real-time PCR (qRT-PCR) using specific primers included in miRNA Assay kit (Life Technologies, Carlsbad, CA) with TaqMan PCR Master Mix (Life Technologies, Carlsbad, CA).

### 2.4. Western Blotting

Total protein was extracted using RIPA buffer (150 mM NaCl, 1% NP-40, 0.5% sodium deoxycholate, 0.1% SDS, and 50 mM Tris, pH 8.0) and protease inhibitors (1 mM PMSF, 10 *μ*g/mL aprotinin, 10 *μ*g/mL leupeptin, and 200 *μ*M sodium orthovanadate). The obtained protein concentration was evaluated in a spectrophotometer. Denatured protein was loaded onto an SDS-PAGE gel and subsequently transferred to a PVDF membrane (Bio-Rad, Hercules, CA). The membrane was incubated with anti-Dnmt3a (1 : 5000, Abcam, Cambridge, UK, #ab13887); anti-Dnmt3b (1 : 800, Santa Cruz Biotechnology, Santa Cruz, CA, #sc-70984); anti-Mecp2 (1 : 1,000, Calbiochem-Merck, Darmstadt, Germany, #472520); or anti-Ezh2 (1 : 2000, Abcam, Cambridge, UK, #ab3748) antibodies. For normalization, the membrane was also incubated with an anti-*β*-actin antibody (1 : 5,000, Santa Cruz Biotechnology, Santa Cruz, CA, #sc-101017). Subsequently, the membrane was incubated with an anti-mouse or anti-rabbit secondary antibody (Life Technologies, Carlsbad, CA) and visualized through chemiluminescence using the Supersignal West Pico Chemiluminescent Substrate (Pierce, Thermo Scientific, Rockford).

### 2.5. Statistical Analysis

Statistical analyses of the gene expression and methylation assays were performed by nonparametric one-way ANOVA, succeeded by Tukey's* post hoc* test, using the GraphPad Prism 5.0 program. At least two independent replicates were performed for each analysis. In all figures, the error bars in the histograms represent the mean standard error of the obtained results.

## 3. Results and Discussion

**Figure 1:**
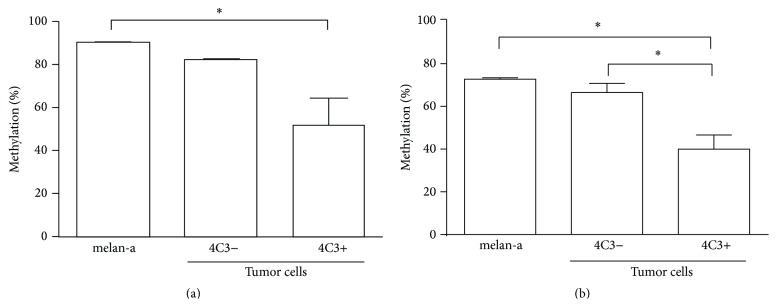
Metastatic melanoma cells are hypomethylated compared with nontumorigenic melan-a cells. The methylation status of three specific CpGs associated with A-repeats (a) and two CpGs in the retrovirus-C repetitive sequence (b) were evaluated using Ms-SNuPE in the melan-a, 4C3−, and 4C3+ cell lines. The percentage of methylation was determined by calculating the average at the different CpG sites analyzed. Ma: nontumorigenic melan-a melanocyte line; 4C3−: nonmetastatic melanoma cell line; 4C3+: metastatic melanoma cell line. ^*∗*^ *p* < 0.05.

**Figure 2:**
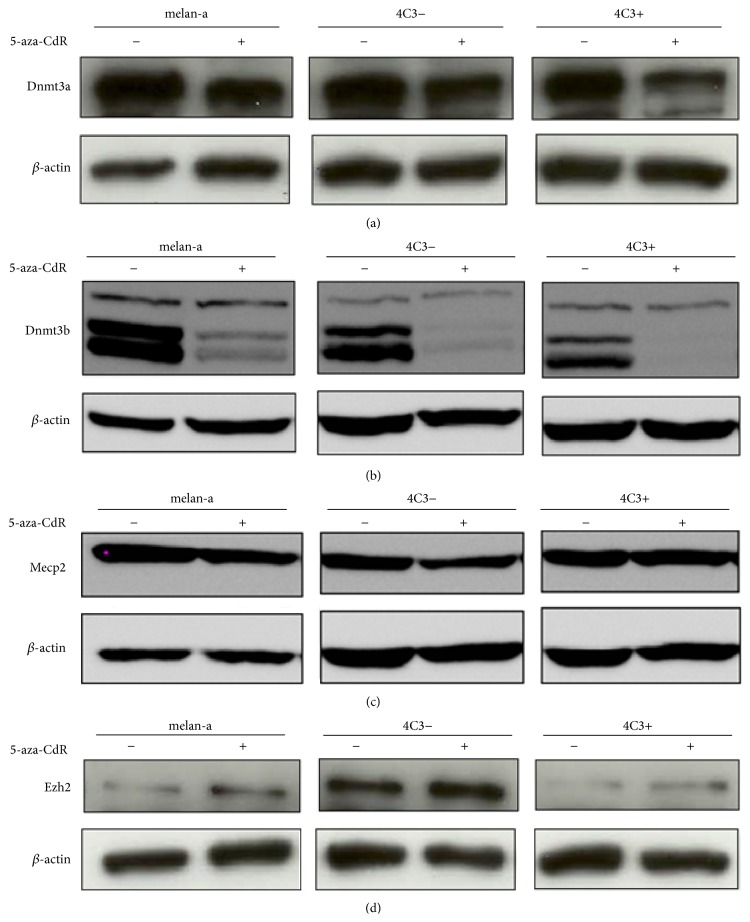
Dnmt3a (a), Dnmt3b (b), Mecp2 (c), and Ezh2 (d) expression profile in melan-a, 4C3−, and 4C3+ before and after treatment with 5-aza-CdR. The protein expression was evaluated by Western blotting in melan-a, 4C3−, and 4C3+ cells that were untreated or treated with 10 *μ*M 5-aza-CdR for 48 hours. The expression of *β*-actin was used for normalization. melan-a: nontumorigenic melan-a melanocyte lineage; 4C3−: nonmetastatic melanoma cell line; and 4C3+: metastatic melanoma cell line.

In a previous study, using a murine melanoma model obtained through sequential anchorage blockade cycles in melan-a melanocytes, Molognoni and coworkers [27] demonstrated alterations in the expression of genes involved in the epigenetic machinery and changes in DNA methylation levels in premalignant melanocytes, suggesting that epigenetic reprogramming occurs prior to malignant transformation in these cells. To verify the contribution of miRNAs to this phenomenon, the expression of key components of the epigenetic machinery was evaluated in parental, melan-a, and melan-a-derived melanoma cells (the nonmetastatic 4C3− cell line and its metastatic derivative, 4C3+). These 4C3 cells have been characterized elsewhere [26, 36]. To verify whether the 4C3− and 4C3+ melanoma cell lines exhibit an altered pattern of DNA methylation, methylation of repetitive DNA sequences (retrovirus-C and intracisternal A particle elements) was assessed using the Ms-SNuPE method. The metastatic 4C3+ cell line showed a significant reduction of global methylation compared with melan-a and nonmetastatic 4C3− cells (Figure 1). This result is consistent with the global demethylation observed in most tumor types [2, 37] and in other melanoma cells in the same murine melanoma model [27]. Hence, the expression of* Dnmt3a, Dnmt3b*,* Mecp2, *and* Ezh2* (Figure 2) of the parental melan-a and melanoma 4C3− and 4C3+ cell lines was assessed at the protein level. Dnmt3b expression was reduced in 4C3− cells compared to normal melanocytes melan-a and even more reduced in 4C3+ metastatic cells (Figure 2(b)). The same pattern was seen in Dnmt3a and Mecp2, however, in an attenuated manner (Figures 2(a) and 2(c)). Taken together, these results show that the global DNA hypomethylation observed during the progression of melanoma is accompanied by downregulation of* Dnmt3a*,* Dnmt3b,* and* Mecp2* expression. Whether DNMT3a and DNMT3b are downregulated during cancer development is a debated issue. Indeed, most tumor types overexpress DNMTs [38]. However, there is evidence that DNMTs are mutated or weakly expressed in cancer cells [39–41]. Our findings are consistent with those of Molognoni et al. [27], who also observed* Mecp2 *downregulation during malignant transformation. However, these authors found that the expression of* Dnmt3b* was not statistically significantly different between melan-a, premalignant, nonmetastatic, and metastatic melanoma cells and* Dnmt3a* was upregulated in the metastatic cell line, possibly reflecting clonal differences. Additionally, we have assessed the expression of* Ezh2* at the protein level (Figure 2(d)) in all 3 studied cell lines. In melan-a and in 4C3+,* Ezh2* was found poorly expressed but overexpressed in 4C3−. Accordingly, this expression fluctuation was also seen in Molognoni et al. [27], showing that Ezh2 might be involved in the primary events of melanocytes transformation.

Recently, it has been reported that numerous miRNAs are involved in the silencing of components of the epigenetic machinery (reviewed in [42]). Moreover, it is been predicted that some miRNAs are involved in the regulation of genes related to the same pathway [43]. Indeed, the miR-29 family is particularly interesting, as overexpression of its members in lung tumor cells significantly reduces* Dnmt3a* and* Dnmt3b* expression [30]. More specifically, miR-29b reduces DNMT1, DNMT3A, and DNMT3B expression in acute myeloid leukemia (AML) [44] and germ cells [45]. miR-29b and miR-29c also target YY1, a chromatin remodeling protein that recruits PRC2 and a histone deacetylase, HDCA, to suppress the expression of specific* loci *[46]. miR-26 is also associated with the downregulation of DNMT3B, since transfection of miR-26b in human breast cancer cells showed diminished expression of DNMT3B. On the other hand, transfection of miR-26b antagomiR showed an overexpression of DNMT3B [32]. miR-26a also targets Ezh2 mRNA [31], and both members of miR-26 family (miR-26a and miR-26b) could target Mecp2/MECP2 mRNA, as predicted by several miRNA target prediction programs (Microrna.org; TargetScan 4.2; Pictar; and DIANA/miRGen). Moreover, miR-203 has been validated as a regulator of* Bmi-1*, which is a member of the Polycomb repressive complex 1 (PRC1) family of proteins and is also involved in epigenetic modifications [47]. Additionally, the expression of miR-203 has been inversed correlated to DNMT3B in breast cancer cells [32, 33]. This miRNA might also target Mecp2/MECP2, as predicted using the same four prediction programs. Thus, these miRNAs are good candidates for the regulation of genes involved in the epigenetic machinery during melanocyte transformation.

**Figure 3:**
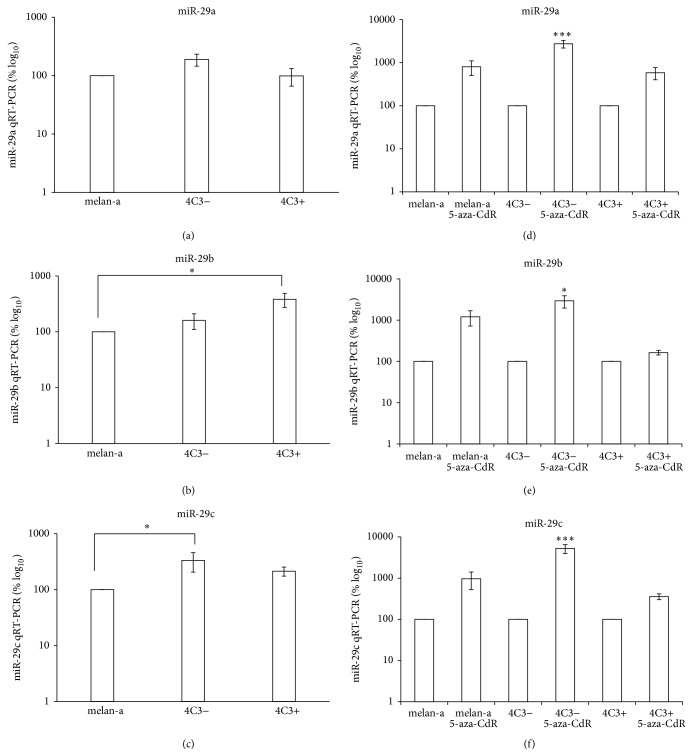
miR-29 expression during melanocyte transformation and 5-aza-CdR treatment. miR-29a (a), miR-29b (b), and miR-29c (c) expression was evaluated in the three cell lines via qRT-PCR. The cells were treated with 10 *μ*M 5-aza-CdR for 48 hours, and the expression of miR-29a (d), miR-29b (e), and miR-29c (f) was subsequently evaluated. U6 was used for normalization. Ma: nontumorigenic melan-a melanocyte lineage; 4C3−: nonmetastatic melanoma cell line; and 4C3+: metastatic melanoma cell line. ^*∗*^ *p* < 0.05; ^*∗∗∗*^ *p* < 0.001.

Since miR-29 family was validated to target Dnmt3a and Dnmt3b [30], the expression profile of the miR-29 family was evaluated and showed that miR-29a did not contribute to the phenotype of the malignant cells, as there were no statistically significant differences observed between the 3 studied cell lines (Figure 3(a)). Nevertheless, miR-29b and miR-29c were significantly upregulated in 4C3+ and 4C3− cells, respectively, compared with melan-a cells (Figures 3(b) and 3(c), resp.). Since* Dnmt3a* and* Dnmt3b* are downregulated in 4C3− and in 4C3+ cells (Figures 2(a) and 2(b)), it is conceivable that these miRNAs regulate the* de novo* Dnmts expression during melanocyte transformation.

**Figure 4:**
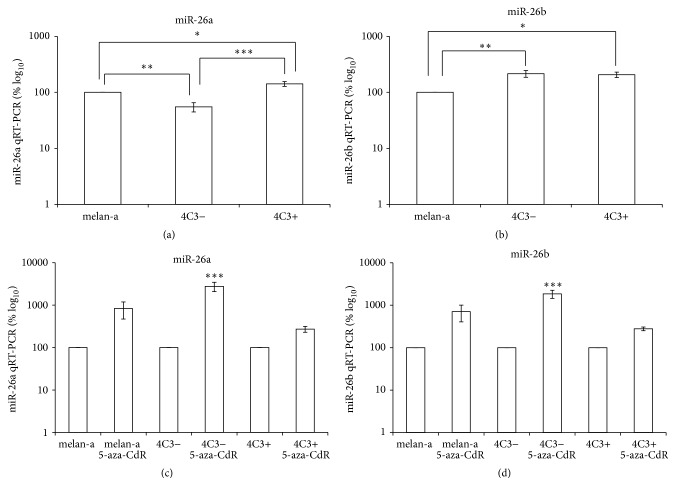
miR-26 expression during melanocyte transformation and 5-aza-CdR treatment. miR-26a (a) and miR-26b (b) expression was examined in the three cell lines using qRT-PCR. The cells were treated with 10 *μ*M 5-aza-CdR for 48 hours, and the expression of miR-26a (c) and miR-26b (d) was subsequently evaluated. U6 was used for normalization. Ma: nontumorigenic melan-a melanocyte lineage; 4C3−: nonmetastatic melanoma cell line; and 4C3+: metastatic melanoma cell line. ^*∗*^ *p* < 0.05; ^*∗∗*^ *p* < 0.01; ^*∗∗∗*^ *p* < 0.001.

The expression of miR-26a was also assessed, showing a significant reduction in its expression in 4C3− cells compared with parental cells. However, upregulation was observed in metastatic melanoma cells compared with melan-a and 4C3− cells (Figure 4(a)). The observed pattern of miR-26a expression is opposed to its validated target Ezh2 (Figure 2(d)). Since the latter is upregulated in nonmetastatic cells compared with melanocytes and metastatic melanoma cells, it is suggestive that miR-26a might regulate Ezh2 expression during the melanocytes malignant transformation. This histone methyltransferase is a part of the Polycomb Complex Repressor 2 (PCR2) system, which represses gene expression through interactions with unmethylated CpG islands in normal cells (reviewed in [48]). Ezh2 has been associated with the aberrant silencing of CpG islands near the promoters of tumor suppressor genes [49]. This site-specific hypermethylation could result from the interaction of Ezh2 with Dnmts to position these proteins near CpG islands, promoting* de novo *methylation (reviewed in [48]). The overexpression of Ezh2 during cell transformation [50] might promote the assembly of Dnmts to the CpG islands providing a more stable silencing mark. Since overexpression of Dnmts is not obligatory to promote aberrant hypermethylation in CpG island in cancer cells [41], low levels of these proteins might be sufficient to silence CpG islands near suppressor gene promoters that are more susceptible to DNA methyltransferases in the presence of abundant Ezh2 [51]. Conversely, downregulation of Dnmt3a, Dnmt3b, and Mecp2 could favor the derepression of repetitive DNA and the global demethylation observed in tumor cells, promoting genomic instability. Thus, overexpression of the epigenetic machinery might not be necessary to maintain the transformed state.

Further, the upregulation of miR-26a seen in 4C3+ cells and miR-26b seen in 4C3− and 4C3+ cells (Figures 4(a) and 4(b)) is opposed to Mecp2 expression in these cells compared with melan-a cells (Figure 2(c)). Because the miR-26 family has been predicted to target Mecp2/MECP2 mRNAs, these miRNAs might be relevant to Mecp2 downregulation and are good candidates to further validation as its regulators. Finally, miR-26b and DNMT3b showed an inverse correlation expression, whereas DNMT3b is downregulated and miR-26b is upregulated in melanoma cells compared to melanocytes, which corroborates to previous evidence that DNMT3b is regulated by miR-26b [32].

**Figure 5:**
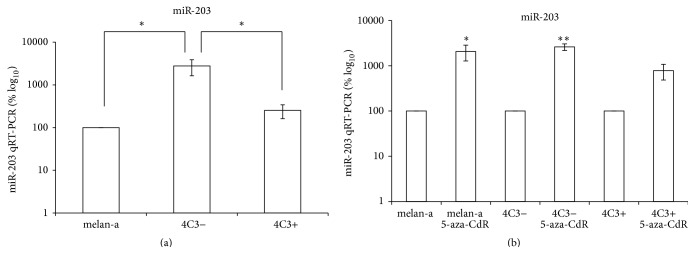
miR-203 expression during melanocyte transformation and 5-aza-CdR treatment. miR-203 (a) expression was evaluated in the three cell lines using qRT-PCR. The cells were treated with 10 *μ*M 5-aza-CdR for 48 hours, and the expression of miR-203 (b) was then evaluated. U6 was used for normalization. Ma: nontumorigenic melan-a melanocyte lineage; 4C3−: nonmetastatic melanoma cell line; and 4C3+: metastatic melanoma cell line. ^*∗*^ *p* < 0.05; ^*∗∗*^ *p* < 0.01.

The analysis of the expression of other miRNA that potentially targets Mecp2 and Dnmt3b, miR-203, revealed that it was significantly upregulated in 4C3− cells compared with the melan-a and 4C3+ cell lines (Figure 5(a)). This finding supports the hypothesis that miR-203 regulates both genes and might be involved in the induction of the transformed state as only the nonmetastatic cell line showed an upregulation of miR-203 expression. Alternatively, miR-203 could be overexpressed in 4C3− cells as a feedback mechanism to avoid transformation, as miR-203 has been previously described as an antimetastatic agent in human prostate cancer [52].

**Figure 6:**
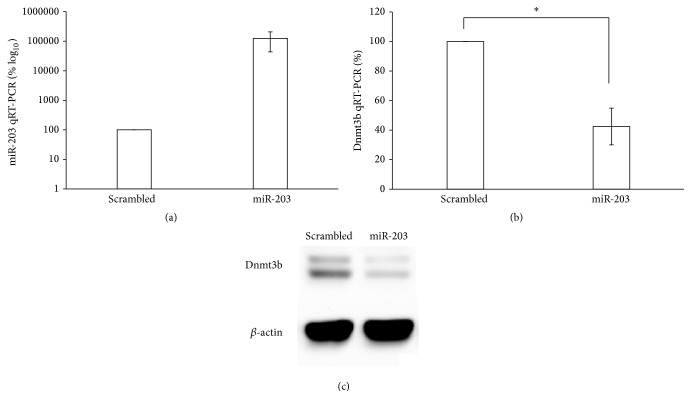
Dnmt3b expression after exogenous overexpression of miR-203 in melan-a cells. (a) miR-203 and (b) Dnmt3b expression was evaluated by qRT-PCR in melan-a cells transfected with pSilencer-mmu-miR-203 or pSilencer-Scrambled. The expression of Dnmt3b at protein level (c) was also evaluated after the same treatment by Western blotting. ^*∗*^ *p* < 0.05.

To test the hypothesis that miR-203 targets Dnmt3b, we overexpress the miR-203 in melan-a cells. A significant downregulation of Dnmt3b at RNA and protein levels was observed (Figure 6), being the first evidence of miR-203 regulating Dnmt3b.

To verify whether these miRNAs could be epigenetically regulated, each cell line was treated with 5-aza-CdR. After treatment, increased miR-203 expression was observed in melan-a and 4C3− cells (Figure 5(b)). These results are consistent with the literature reporting that miR-203 is epigenetically silenced in human tumor cells [53, 54]. miR-26 and miR-29 family members were also overexpressed in 4C3− cells compared with untreated cells (Figures 4(c), 4(d), 3(d), 3(e), and 3(f), resp.). Given that miR-26 and miR-29 exhibit expression patterns similar to that of miR-203 in 4C3− cells treated with 5-aza-CdR, it is conceivable that these miRNAs are also epigenetically regulated in these cells. The expression of these miRNAs was not statistically significantly different in treated metastatic 4C3+ cells compared with untreated 4C3+ cells, possibly because the untreated counterpart cells are already demethylated. An* in silico* analysis of CpG islands near the miRNA genes of* Mus musculus* and their promoters was performed using Genome Browser (http://genome.ucsc.edu/cgi-bin/hgGateway) and the Transcriptional Regulatory Element Database (http://rulai.cshl.edu/cgi-bin/TRED/tred.cgi?process=home).  The analysis showed that* miR-26a-1* is hosted within a gene, whose promoter overlaps with a CpG island of 1230 bp. The intragenic miRNAs,* miR-26a-2 *(*miR-26a-2 *and* miR-546*) cluster and* miR-26b, *are both associated with CpG islands of 1752 and 1098 bp near the transcription start site (TSS) in the host gene or overlapping with the predicted host gene promoter, respectively. The* miR-29a/29b-1* cluster is intergenic and exhibits 4 small CpG islands near the TSS, ranging from 206 to 836 bp. It has been predicted that this cluster is imprinted, as it is embedded into the imprinted* Copg 2* cluster [55]. The only exception to this pattern is the intragenic* miR-29b-2/29c* cluster, which presents a 558 bp CpG island distant from its TSS. miR-203, by its turn, is intergenic and seems to be embedded in a CpG island of 804 bp. These analyses showed that all miRNAs studied here are near of regions commonly regulated epigenetically.

To our knowledge, this study provides the first evidence that* miR-26a*,* miR-26b*,* miR-29b,* and* miR-29c *might be epigenetically regulated in mouse and is in agreement with study of Desjobert and colleagues that showed* miR-29a* methylated in human lymphoma cells [56]. An estimated 60% of human coding genes have been associated with CpG islands, while approximately half of known miRNA genes are located near or embedded within a CpG island, indicating that methylation is an important mechanism for the regulation of miRNA transcription [57]. However, upregulation of miRNAs following 5-aza-CdR treatment was observed only in the nonmetastatic melanoma cell line, potentially reflecting the unstable epigenetic marks in these cells. The expression of Dnmt3b was assessed from total protein extracts, demonstrating the efficiency of 5-aza-CdR treatment in inducing Dnmt3b degradation (Figure 2(b)). In contrast, no differences in Mecp2 expression were observed at the protein (Figure 2(c)) following 5-aza-CdR treatment compared with untreated cells. Taken together, these findings support the idea that these miRNAs are epigenetically regulated. Nevertheless, they must be further characterized.

In conclusion, this study demonstrated that miR-203 overexpression promotes Dnmt3b downregulation. Additionally, it highlights the importance of miR-26, miR-29, and miR-203 in promoting the imbalanced expression of genes involved in the epigenetic machinery during the malignant transformation of melanocytes. These results indicate that the miRNA methylation status plays an important role in the establishment of the altered epigenetic state observed in tumors.
